# Postcopulatory sexual selection influences baculum evolution in primates and carnivores

**DOI:** 10.1098/rspb.2016.1736

**Published:** 2016-12-14

**Authors:** Matilda Brindle, Christopher Opie

**Affiliations:** Department of Anthropology, University College London, 14 Taviton Street, London, WC1H 0BW, UK

**Keywords:** baculum, postcopulatory sexual selection, prolonged intromission, primates, carnivores, Bayesian phylogenetics

## Abstract

The extreme morphological variability of the baculum across mammals is thought to be the result of sexual selection (particularly, high levels of postcopulatory selection). However, the evolutionary trajectory of the mammalian baculum is little studied and evidence for the adaptive function of the baculum has so far been elusive. Here, we use Markov chain Monte Carlo methods implemented in a Bayesian phylogenetic framework to reconstruct baculum evolution across the mammalian class and investigate the rate of baculum length evolution within the primate order. We then test the effects of testes mass (postcopulatory sexual selection), polygamy, seasonal breeding and intromission duration on the baculum in primates and carnivores. The ancestral mammal did not have a baculum, but both ancestral primates and carnivores did. No relationship was found between testes mass and baculum length in either primates or carnivores. Intromission duration correlated with baculum presence over the course of primate evolution, and prolonged intromission predicts significantly longer bacula in extant primates and carnivores. Both polygamous and seasonal breeding systems predict significantly longer bacula in primates. These results suggest the baculum plays an important role in facilitating reproductive strategies in populations with high levels of postcopulatory sexual selection.

## 1. Introduction

The morphology of male intromittent organs is argued to be subject to more rapid divergent evolution than any other form in the animal kingdom [1]. The baculum, or penis bone, does not buck this trend and has been described as ‘the most diverse of all bones’ ([2], p. 1), varying dramatically in length, width and shape across the Mammalia.

The baculum is not uniformly present across mammals. It was thought only to exist in eight of the mammalian orders: Afrosoricida, Carnivora, Chiroptera, Dermoptera, Erinaceomorpha, Primates, Rodentia and Soricomorpha [3,4]. However, it has recently been discovered that a Lagomorph, the American pika (*Ochonta princeps*), also has a small baculum [5]. This discovery suggests that baculum presence may be more prevalent across mammals than historically assumed. Certain orders have a mixture of baculum presence and absence across species; these are the Carnivora, Chiroptera, Primates and Rodentia. In Primates, for example, humans, tarsiers and several Platyrrhines lack a baculum. The Lagomorphia may be similarly divided. Aside from documenting the presence and absence of the baculum across the mammalian orders, the evolutionary history of the baculum had not been studied until recently, leaving many questions unanswered.

Genital (and hence bacular) morphology is suggested to be subject to sexual selection [6]. The few empirical tests conducted to date may support this hypothesis. Stockley *et al.* [7] found that baculum width in polygamous house mice (*Mus domesticus*) was a significant predictor of male reproductive success. Simmons & Firman [8] were able to manipulate baculum width experimentally by altering the level of sexual selection pressure in populations of house mice. After 27 generations, populations with artificially enforced high levels of postcopulatory sexual selection pressure had significantly thicker bacula than populations in which monogamy was enforced and sexual selection pressure was therefore absent. These studies indicate that intra-sexual selection, in particular postcopulatory sexual selection pressure, may be driving bacular evolution. If this is the case, the bacula of populations under high levels of intra-sexual selection pressure, such as those with polygamous (multi-male, multi-female) or seasonal mating systems, should be subject to stronger evolutionary forces.

Different mating systems generate variation in levels of postcopulatory sexual selection pressure and therefore morphological variability; for example, species with high levels of sperm competition tend to have large testes relative to their body mass [9]. Residual testes mass is thus considered to be a reliable measure of the mating system of a population and therefore the degree of sexual selection pressure [9]. Orr & Brennan [10] found that relative testes mass was a significant predictor of baculum presence across Chiroptera, Eulipotyphla, Primates and Rodentia. However, the same study found no relationship between baculum presence or width and mating system, indicating that a third variable may be at play. Ramm [11] tested for a relationship between testes mass and baculum length in four orders by first establishing the level of phylogenetic dependency between species and then conducting appropriately corrected regressions. A positive relationship between testes mass and baculum length was noted in Rodentia and Carnivora, but the same test found no relationship in Primates or Chiroptera.

The adaptive function of the baculum, under high levels of intra-sexual selection, is yet to be established. A potential strategy by which a male could increase their reproductive success, by outcompeting rival males, is through prolonging intromission and consequently delaying a female mating with another male [12]. The prolonged intromission hypothesis argues that the baculum helps to facilitate this prolonged duration of intromission by supporting the penis [13].

In this context, the proximate mechanism of the baculum is to act as a supportive rod, strengthening the penis and protecting the urethra during prolonged intromission [12]. A recent study on three different species of bat found that the baculum formed a functional unit with the *corpora cavernosa*, which protected the glans tip and the shaft of the penis when erect [14]. The authors posit that the baculum also helps to limit constriction of the distal urethra and urethral opening in the erect penis during intromission, facilitating sperm flow. In many species of primate in which the baculum is elongated, the distal end of the bone projects slightly from the urethra while the penis is erect [15]. This could bring the baculum into contact with a female's cervix during intromission, facilitating the transfer of semen into the cervical canal [12,15].

Evidence for the prolonged intromission hypothesis has so far proven controversial. Early studies found that prolonged intromission was correlated with elongated bacula in primates and carnivores [13,16]; however, these studies did not take into account the statistical non-independence of data that arises due to a shared evolutionary history between species. A later study, corrected to account for phylogeny, tested for a correlation between prolonged intromission and baculum length in North American carnivores, but did not find support for the hypothesis [17]. However, data to test this hypothesis were only available for 18 species, of which only two were characterized as having short intromission duration. Dixson *et al.* [18] argue that this sample is not representative enough to decisively refute the prolonged intromission hypothesis, and carried out their own phylogenetically corrected analysis in a sample of 57 species of mammal. This time, a significant correlation was found between the two variables. Although both studies were corrected for phylogeny, if the degree of phylogenetic dependency is not established before an analysis is adjusted, correcting for phylogeny can produce misleading or incorrect results, as the level of relatedness between species varies across a phylogeny [19]. Bayesian Markov chain Monte Carlo (MCMC) analyses enable species' phylogenies to be incorporated into an analysis, rather than simply correcting for phylogeny, and can thus produce more reliable results [20,21].

In a new study, Schultz *et al.* [22] used the earlier phylogenetic methods of stochastic mapping to model the presence and absence of the baculum in 954 mammalian species, and argue that the baculum independently evolved a minimum of nine times in mammals. However, this sample is unlikely to reflect the course of evolution across the entire mammalian class, particularly as baculum absence was only noted in 103 species.

Here, we use phylogenetic comparative methods within a Bayesian MCMC framework [23] to examine the evolutionary history of the baculum and investigate the hypothesis that increased levels of intra-sexual selection affect baculum evolution. We first reconstructed the evolutionary trajectory of the baculum across the entire mammalian class and examined the rate of bacular evolution in the primate order. Then, we tested for a relationship between baculum length and testes mass in both primates and carnivores. We then further tested for correlated evolution between baculum presence and intromission duration in primates, before conducting phylogenetic *t*-tests to establish whether primates and carnivores with prolonged intromission durations have longer bacula than those with short intromission durations. Finally, we used the same tests to examine whether increased levels of postcopulatory sexual selection pressure caused by (i) polygamous mating systems and (ii) seasonal breeding patterns led to an increase in baculum length in primates. Primates and carnivores are likely to be particularly rewarding groups to study, because there is a mixture of baculum presence and absence within each order. This means that differences between those with and without bacula can be tracked at the species level, rather than across orders. Furthermore, as these orders are arguably more extensively studied than other mammalian groups, there are more data available.

If the baculum facilitates prolonged intromission and increased proximity to the cervix in order to reduce the level of sperm competition and increase reproductive success, then several predictions can be made and tested. It would be expected that there would be a relationship between baculum length and testes mass, which can be used as a proxy for the level of postcopulatory sexual selection pressure in a population. Intromission duration would be expected to correlate with baculum presence across the course of evolution. Species with prolonged intromission durations should have elongated bacula. Finally, groups in which postcopulatory sexual selection pressure is highest, such as those in which mating is polygamous or occurs seasonally, should have longer bacula than groups with lower levels of postcopulatory sexual selection pressure.

## 2. Material and methods

Ancestral state reconstructions, tests of correlated evolution, tests of trait relationships and phylogenetic *t*-tests were all conducted using BayesTraits (v. 2) [24]. Trait data were compiled from the literature (electronic supplementary material).

A supertree phylogeny of 5020 extant mammals was used to reconstruct the ancestral states of baculum presence across the mammalian order [25]. All analyses of the primate and carnivore orders were conducted on a posterior distribution of 10 000 molecular phylogenies inferred using Bayesian MCMC methods [26]. Chronograms were used in all ancestral state reconstructions and tests of correlated evolution, whereas phylograms were used in tests of trait relationships and phylogenetic *t*-tests.

A reversible-jump MCMC method with an exponential hyperprior ranging between 0 and 0.07 was used to estimate discrete ancestral states [27]. Each chain was run for 5 million iterations with a burn-in of 50 000 iterations; this was the case for all analyses aside from the variable-rates model. We were interested in inferring seven key nodes across the mammalian phylogeny, as well as the ancestral state of baculum presence for both the primate and carnivore orders (electronic supplementary material, figure S1). Nodes were constructed using the ‘add MRCA’ procedure in BayesTraits [24].

A variable-rates model was used to reconstruct the course of baculum length evolution across the primate order (following Venditti *et al.* [28]). The model allows the rate of evolution to change across a phylogeny over time, identifying when and where evolutionary rates have differed without prior knowledge. Stretched branches of the phylogenetic tree indicate that a trait has evolved quickly and compressed branches indicate slow rates of trait change. The model was run for 10 million iterations, with a burn-in of 100 000 iterations.

Baculum length and testes mass were tested for a relationship using a multiple regression between baculum length, testes mass and adult male body mass. The model was run three times and the chain with the median log marginal likelihood was chosen; this approach was also taken when conducting correlated evolution and hypothesis tests. The proportion of the slope parameter (*β*) that crossed zero was used to establish the *p*-values (following Organ *et al.*'s [29] method for phylogenetic *t*-tests).

To test for correlated evolution between baculum presence and intromission duration, we compared the log marginal likelihood of independent (traits constrained to evolve separately) and dependent models. An exponential hyperprior with a range of 0–0.05 was used. The two models were compared using log natural Bayes factors (BFs), calculated as two times the difference in log marginal likelihood between the models [24]. BFs were interpreted following Kass *et al*. [30]: 0–2, minimal support; 2–6, positive support; 6–10, strong support; more than 10, very strong support. Recent literature has highlighted several issues with using the harmonic mean as a measure for estimating the log marginal likelihood of a model and the relative merits of the stepping-stone sampling method, which is argued to be more accurate [31,32]. We therefore used the stepping-stone sampling method to estimate the log marginal likelihood. One hundred stones were used per 10 000 iterations of the Markov chain. Following Xie *et al*. [32], a beta (*α*, 1.0) distribution was employed and *α* was set at 0.3. Stepping-stones were used in this way for all tests of trait relationships, correlated evolution and phylogenetic *t*-tests.

MCMC phylogenetic *t*-tests were used to test hypotheses accounting for the statistical non-independence of the data, due to shared evolutionary history (following Organ *et al*. [29]).

## 3. Results

A multistate analysis in BayesTraits [24] (*n* = 1818) indicates that the ancestral mammal did not possess a baculum (baculum absence, mean probability = 0.98). Ancestral state reconstructions across six nodes (table 1; electronic supplementary material, figure S1) suggest that the baculum first evolved after non-placental and placental mammals split (baculum absence, mean probability = 0.93), but before the most recent common ancestor (MRCA) of primates and carnivores evolved (baculum presence, mean probability = 0.99). The ancestral primate and carnivore both had a baculum (baculum presence, mean probability = 1.00 and 1.00, respectively).

**Table 1.** Probability of baculum presence or absence at the root and six nodes of the mammalian phylogeny (see electronic supplementary material, figure S1, for nodes). Probabilities of baculum presence or absence of ancestral primates and carnivores are also given.

	baculum absent		baculum present	
	mean probability	s.e.	mean probability	s.e.
root	0.98	0.0011	0.02	0.0011
node 1	0.93	0.0020	0.07	0.0020
node 2	0.51	0.0016	0.49	0.0016
node 3	1.00	0.0000	0.00	0.0000
node 4	0.01	0.0000	0.99	0.0000
node 5	0.3	0.0010	0.7	0.0010
node 6	0.01	0.0000	0.99	0.0000
primates	0.00	0.0000	1.00	0.0000
carnivores	0.00	0.0000	1.00	0.0000

**Figure 1.**
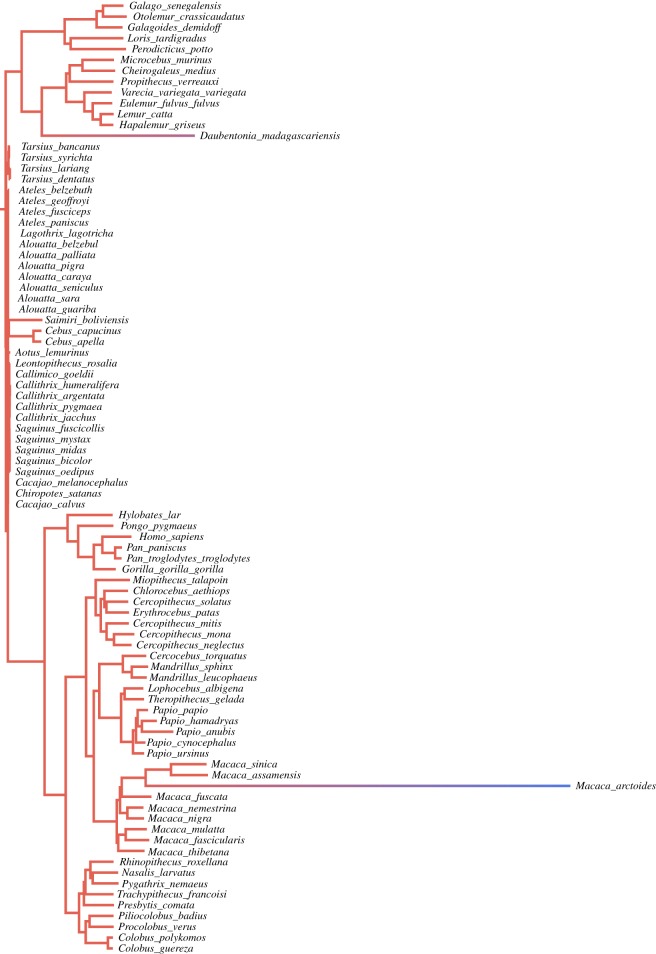
A primate phylogeny scaled to reflect the rate of bacular evolution. Darker red branches indicate lower rates of evolution; blue branches indicate particularly high rates of evolution.

The evolutionary trajectory of baculum length was visualized using the variable-rates model, which stretches or shrinks the branches of a phylogenetic tree according to differing rates of trait evolution (figure 1; electronic supplementary material, figures S2–S4). Primates show marked variation in baculum length and this is reflected in their evolutionary history. Very little change has occurred in platyrrhines and tarsiers, which tend to have small or absent bacula, indicating that the baculum of the ancestral primate was fairly small. By contrast, strepsirrhines and catarrhines have undergone relatively fast evolutionary change, and generally have longer bacula than the platyrrhines and tarsiers. The apes represent an interesting group as they have undergone high rates of change, yet have very small or absent bacula. This suggests that after the platyrrhine and catarrhine lineages split, the baculum of the ancestral catarrhine underwent a high rate of evolution and became a lot longer. When apes subsequently split from Old World monkeys this trend reversed and the ape baculum underwent further high rates of evolution, this time reducing in length. (See electronic supplementary material for variable-rates trees depicting carnivore baculum length evolution and primate and carnivore testes mass evolution, figures S2–S4.)

Baculum length could not be predicted from testes mass in either primates (*n* = 46, *p* = 0.139, *R*^2^ = 0.03) or carnivores (*n*= 32, *p*= 0.231, R^2^= 0.37) (electronic supplementary material, table S2).

**Figure 2.**
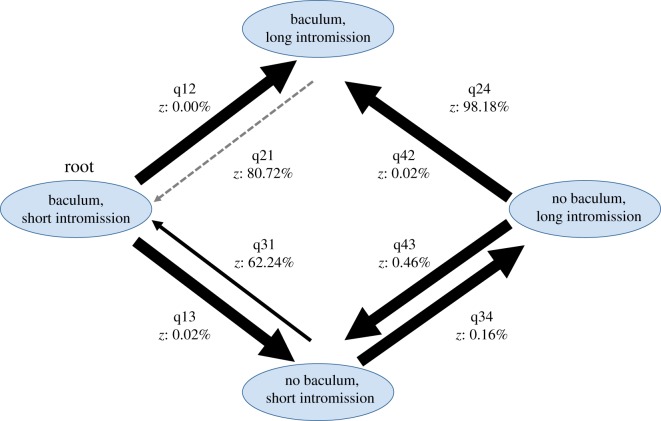
Coevolution between primate baculum presence and intromission duration. *z* percentages show the posterior probability that a transition rate from one state to another is zero (i.e. how often a given transition does not occur). Thick black arrows indicate that a transition happened frequently; thinner or absent arrows indicate that a transition was rare or practically non-existent. (Online version in colour.)

We found positive evidence for correlated evolution between baculum presence and intromission duration in primates (*n* = 299, log BF = 4.78; table 2). Ancestral state reconstructions and model rates indicate that baculum presence and short intromission durations (mean probability = 0.73) preceded a shift to prolonged intromission (figure 2). After long intromission durations had evolved, the baculum was rarely lost. However, the baculum was often lost if intromission duration remained short. Long intromission durations rarely became short again when a baculum was present. By contrast, when a baculum was absent, intromission duration switched frequently between being long and short.

**Table 2.** Likelihood of dependent and independent models of correlated evolution between baculum presence and intromission duration in primates. The Bayes factor indicates positive support for the dependent model of evolution over the independent model. Bayes factors were interpreted following Kass *et al.* [30]: O–2, minimal support; 2–6, positive support; 6–10, strong support; more than 10, very strong support.

	dependent model	independent model	
coevolution analysis	log likelihood	log likelihood	log natural Bayes factor
intromission duration	−45.77	−48.16	4.78

The hypothesis that postcopulatory sexual selection influences baculum length was tested through a series of phylogenetic *t*-tests (table 3). In line with our predictions, we find that species in both the primate and carnivore orders in which intromission is prolonged have significantly longer bacula than species in which intromission is short (*n* = 53, *p* = 0.000 and *n* = 41, *p =* 0.018, respectively). Primates in polygamous mating systems were found to have significantly longer bacula than those in other mating systems (*n* = 65, *p* = 0.032). Finally, seasonally breeding primates have significantly longer bacula than primates that do not breed seasonally (*n* = 63, *p* = 0.045).

**Table 3.** Phylogenetic *t*-tests of baculum length and intromission duration in primates and carnivores, and mating system and breeding seasonality in primates.

	primates			carnivores		
model	*β*	s.e. *β*	*p*-value	*β*	s.e. *β*	*p*-value
baculum length and intromission duration	16.64	±0.05	*p* = 0.0000	64.90	±0.41	*p* = 0.018
baculum length and mating system	−3.45	±0.03	*p* = 0.0318	—	—	—
baculum length and breeding seasonality	−3.55	±0.03	*p* = 0.0448	—	—	—

## 4. Discussion

Our results have uncovered the evolutionary trajectory of the baculum across the mammalian class, showing that the baculum first evolved after placental and non-placental mammals split around 145 million years ago (Ma), but before the MRCA of primates and carnivores evolved around 95 Ma [25]. We show for the first time that both the ancestral primate and the ancestral carnivore had a baculum, a result bearing important implications for how the baculum should be studied within these orders. Analyses should focus on examining why the baculum was retained in certain species and lost in others, not why the baculum might have evolved; it was already present in their ancestors.

We found no relationship between baculum length and testes mass in primates or carnivores, supporting previous findings in primates, but not carnivores [11]. This discrepancy is probably explained by the use of a Bayesian phylogenetic framework for our analyses; our finding suggests that any observed relationship between baculum length and testes mass could have evolved by chance. Although these results do not provide support for the hypothesis that baculum length is sexually selected, they do not refute it. It is possible that aspects of baculum morphology, such as width or shape, are more likely to vary with testes mass. For instance, baculum shaft width is a significant predictor of the number of offspring sired by male house mice [7]. Indeed, Orr & Brennan found that testes mass predicted baculum width in four orders of mammal; however, when this relationship was tested using a phylogenetic model, baculum width was no longer a significant predictor [10]. It is possible that these results would have remained significant if the relationship had been examined at the order level, as bacular function may vary from order to order. Our results serve to highlight the importance of using full phylogenetic methods when examining trait evolution.

This study has been the first to demonstrate that baculum presence has correlated with intromission duration over the course of primate evolution. The result highlights the interplay between morphological and behavioural phenotypes over evolutionary time. The baculum physically supports and protects the male's penis [12,14], and assists the transfer of semen towards a female's cervix [12,15]. However, it also plays an important role in facilitating prolonged intromission, which itself may be a sexually selected behaviour, aimed at increasing reproductive success by delaying females from re-mating [12].

Our results confirm that the prolonged intromission hypothesis remains robust when analysed within a rigorous phylogenetic framework. Phylogenetic *t*-tests show that the baculum is significantly longer in both primate and carnivore species in which intromission is prolonged. This suggests that the elongation of the baculum over the course of mammalian evolution was probably driven by its utility in prolonged intromission. Two more phylogenetic *t*-tests showed that primates in polygamous mating systems and seasonally breeding primates had significantly longer bacula than primates in other mating systems and those without a seasonal breeding pattern, highlighting the importance of postcopulatory sexual selection as a driver of bacular evolution.

The finding from the test of correlated evolution, coupled with the results of the phylogenetic *t*-tests, allows us to begin piecing together the proximate and ultimate functions of the baculum. Polygamous mating systems and limited breeding seasons create high levels of postcopulatory sexual competition. In this environment, prolonging intromission could delay a female from re-mating, thus increasing a male's chance of successfully fertilizing her under competitive conditions. Ensuring that the urethra is unrestricted and there is as little distance as possible for sperm to travel is a way of increasing the amount of sperm transported to the cervical canal. The baculum serves as a supportive structure during prolonged intromission, both protecting the urethra and preventing it from being constricted [14].

These results do not necessarily apply to other orders within the mammalian class, but they do highlight potentially rewarding lines of enquiry. It is also important to note that, even within the primate and carnivore orders, other factors are likely to influence whether a baculum is retained or not and how its morphology evolves. Studies of bacular evolution tend to focus on why it is present in certain species, or why it might have increased in length or width; factors driving the reduction or disappearance of bacula have largely been ignored.

Ingenious studies are beginning to pick apart the proximate mechanism of the baculum, as well as some of the factors driving its evolution in different mammalian orders [8,14]. By comparing these findings across orders and examining the baculum through a phylogenetic framework, we can begin to build a more comprehensive picture of the proximate and ultimate functions of the baculum, and how it evolved in extant species and their ancestors.

## Supplementary Material

Brindle_Opie_tables_figures_ESM

## Data accessibility

Data are available from Dryad Digital Repository [33].
