# Supplementary material for: Postcopulatory sexual selection influences baculum evolution in primates and carnivores
Source: Proc Biol Sci. 2016 Dec 14;283(1844):20161736. doi: 10.1098/rspb.2016.1736 (PMC5204150; doi:10.1098/rspb.2016.1736)
Supplement: Brindle_Opie_tables_figures_ESM [file rspb20161736supp1.pdf]

Electronic Supplementary Material

Postcopulatory sexual selection influences baculum evolution in primates and carnivores

Matilda Brindle and Christopher Opie

Proceedings of the Royal Society B: Biological Sciences (DOI 10.1098/rspb.2016-1736)

These supplementary materials provide information additional to that contained in the main paper on: trait data, ancestral state reconstructions using additional data and the variable rates model, and other analyses.

Notes on Trait Data

a) Baculum data

When more than one data point was available, the mean baculum length was used.

b) Body mass data

All body masses are those of adult males, aside from species in which no body size dimorphism exists.

c) Intromission data

'Long' intromission was defined as that which continued for longer than 180 seconds and 'short' intromission was defined as that which continued for less than 180 seconds (following Dixson [1]).

d) Mating system data

Primates for which more than one mating system was cited in the literature were coded as such. If the polymorphism included polygamy, the species was removed from the dataset before analyses were run as this effectively meant it fell on both sides of the binary grouping variable ('polygamy' versus 'other').

e) Breeding seasonality data

Primates were classed as 'seasonal breeders' if over 67% of births occurred over a peak three-month period [2].

Mammalian Ancestral State Reconstructions

Species within the orders Pholidota and Xenarthra have been consistently excluded from literature describing the mammalian orders in which the baculum is present, suggesting that a baculum is not present in these orders. However, since it was not possible to find a source explicitly stating that this was the case, these orders were excluded from our main reconstruction of the evolution of the baculum across mammals. Similarly, species within the orders Afrosoricida and Eulipotyphla have been consistently defined as orders in which a baculum is present and it has not been possible to find any indication that this is not the case for all species within the order [3,4]. However, since no source has directly stated that all species within these orders have a baculum, they too were excluded from our main reconstruction of bacular evolution.

This approach, although cautious, may not be as accurate as if these orders were to be included. For this reason, we re-ran exactly the same multistate analysis across the mammalian phylogeny, this time including Pholidota, Xenarthra, Afrosoricida and Eulipotyphla. The results of this analysis (table S1) produce a more specific time frame for the first evolution of the baculum, suggesting an initial appearance after node 2, but before node 4 (see figure S1). Another notable change in this version of the analysis is that the baculum is indicated to be absent at node 5, rather than present as in the original analysis. This is understandable given the addition of an entire order in which a baculum is absent to this clade.

Table S1. Probability of baculum presence or absence at the root and seven nodes of the mammalian phylogeny.

	Baculum Absent		Baculum Present	
	**Mean Probability**	**Standard Error**	**Mean Probability**	**Standard Error**
**Root**	1.00	0.0000	0.00	0.0000
**Node 1**	1.00	0.0001	0.00	0.0001
**Node 2**	0.92	0.0005	0.08	0.0005
**Node 3**	1.00	0.0000	0.00	0.0000
**Node 4**	0.00	0.0000	1.00	0.0000
**Node 5**	0.89	0.0005	0.11	0.0005
**Node 6**	0.01	0.0000	0.99	0.0000
**Node A**	0.03	0.0000	0.97	0.0000

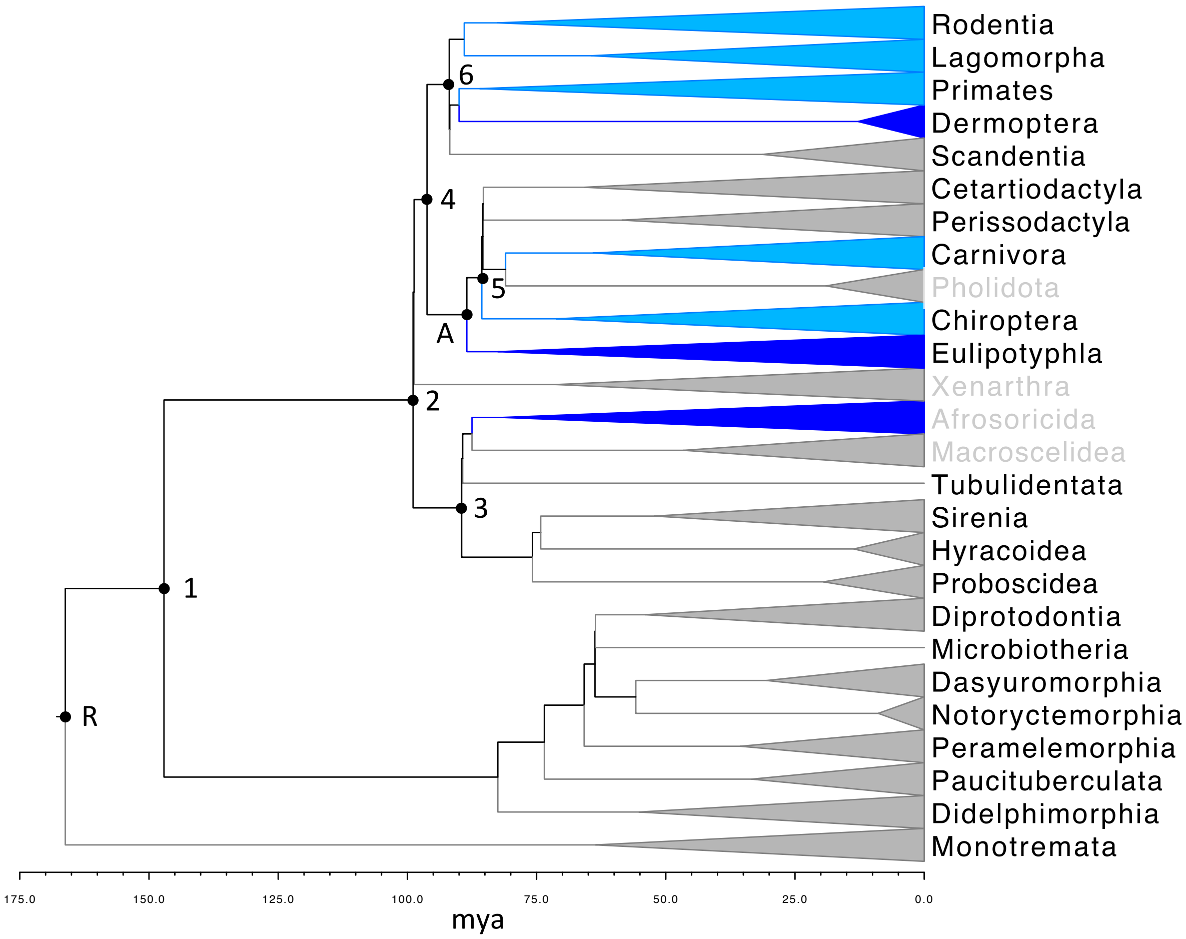

Figure S1. Mammal phylogeny [5] with the ancestral nodes (R = root, nodes 1-6) used for reconstruction (table 1, table S1). Node A is only relevant when the orders Eulipotyphla and Pholidota are included in the analyses (table S1). Orders are colour coded: grey = no species with bacula, blue = all species have bacula, light blue = mixed, some species with bacula. Taxa names are grey if they were excluded from the main analyses (table 1), but are included in the broader analysis (table S1).

Ancestral State Reconstructions: variable rates model

A single time-calibrated phylogenetic tree is required to use the variable rates model. Tree Annotator, a programme available in the BEAST software package [6], was used to create a single maximum clade credibility (MCC) tree from the block of 10,000 trees used in the variable rates analyses [7].

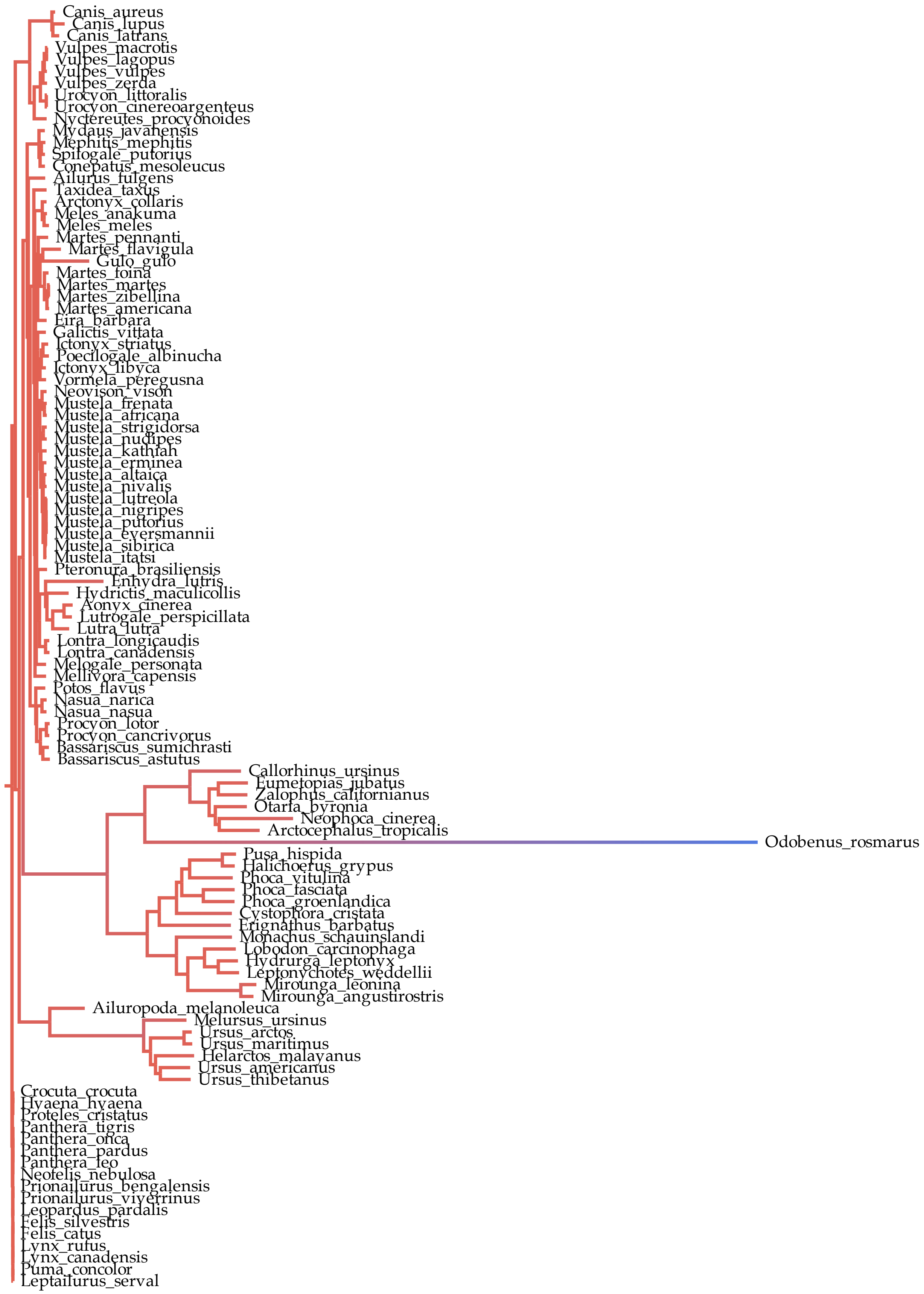

Figure S2. A carnivore phylogeny [7] scaled to reflect the rate of bacular evolution [8]. Darker red branches indicate lower rates of evolution; blue branches indicate particularly high rates of evolution.

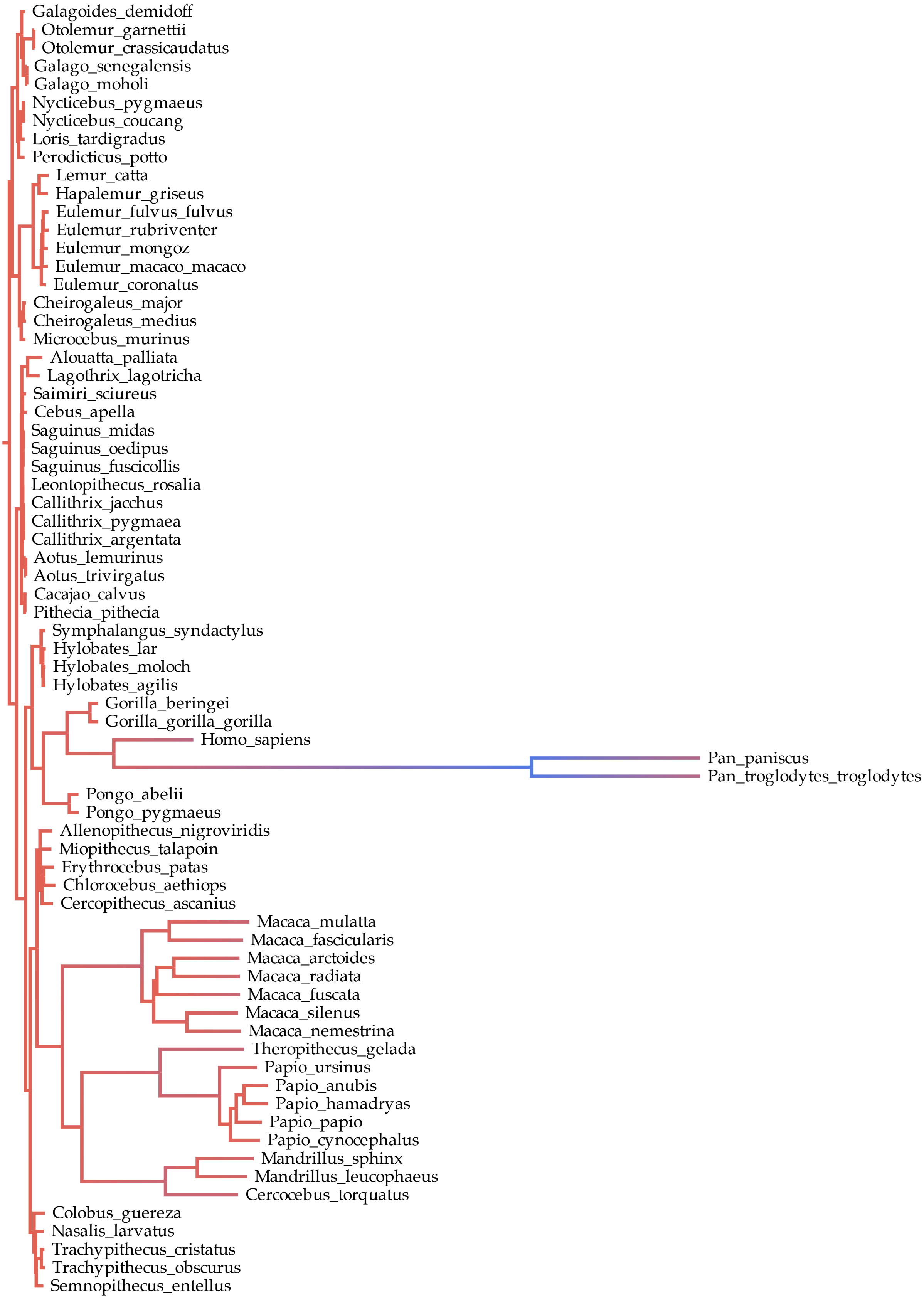

Figure S3. A primate phylogeny [7] scaled to reflect the rate of testes evolution [8]. Darker red branches indicate lower rates of evolution; blue branches indicate particularly high rates of evolution.

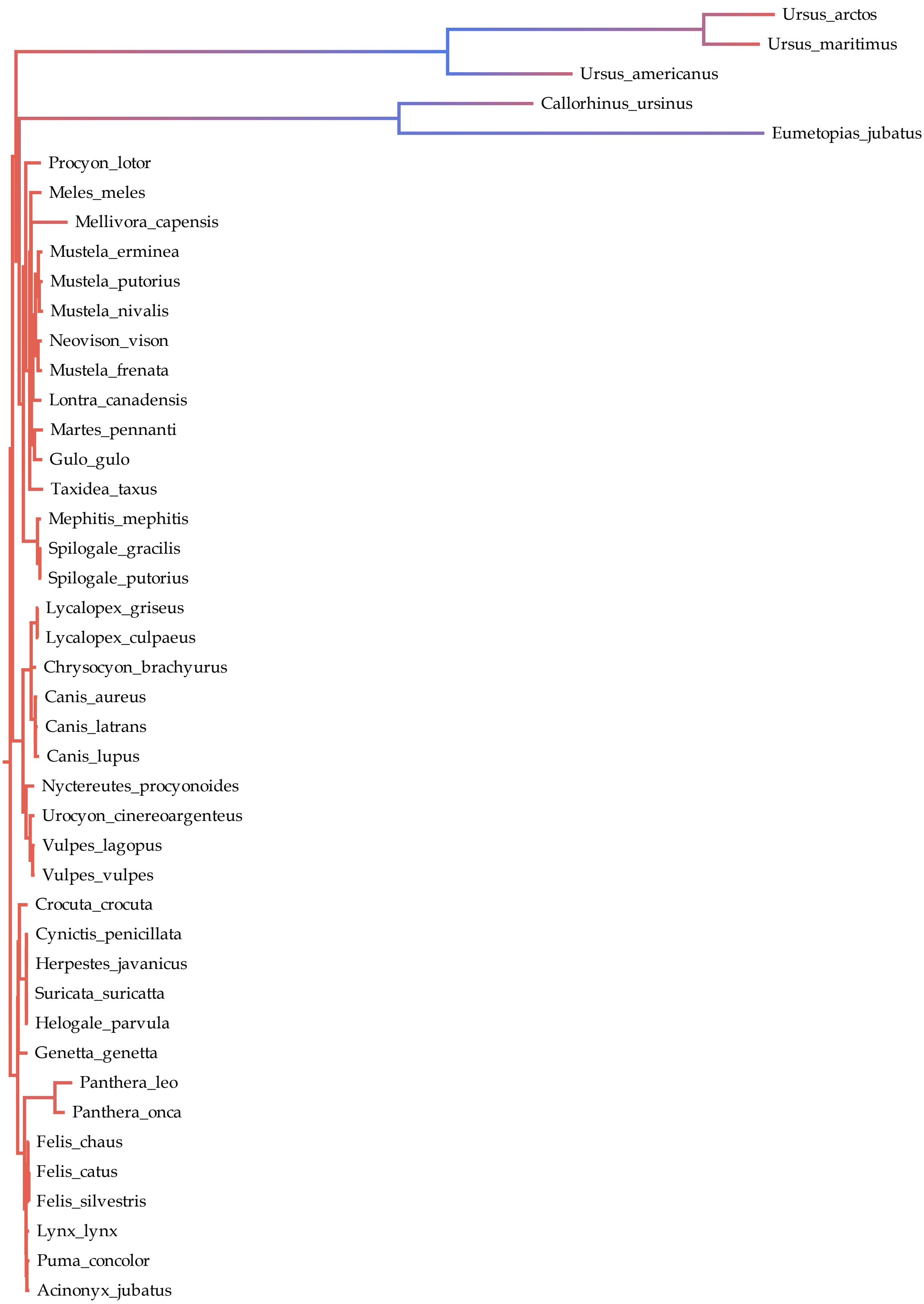

Figure S4. A carnivore phylogeny [7] scaled to reflect the rate of testes evolution [8]. Darker red branches indicate lower rates of evolution; blue branches indicate particularly high rates of evolution.

Continuous Regression

Table S2. Phylogenetic regression between baculum length and testes mass in primates and carnivores.

Model	Primates			Carnivores		
	Log likelihood	*p*-value	R^2	Log likelihood	*p*-value	R^2
Baculum length and testes mass	-192.08	p=0.14	0.03	-173.99	p=0.23	0.37

These analyses suggest that testes mass (a proxy for postcopulatory selection pressure) is not a significant predictor of baculum length in primates or carnivores.

Correlated Evolution

It was not possible to test for correlated evolution between baculum presence and intromission duration in carnivores. Since the Hyaenidae family are the only carnivores in which a baculum is completely absent, the sample was not large enough to produce meaningful results. For this reason, carnivores are only included in analyses examining baculum length.

Hypothesis Testing

Species were divided into groups by creating binary variables and regressing baculum length onto these (following Organ *et al.* [9]). Adult male body mass was included as a predictor variable in order to account for its effect on baculum length.

Unfortunately it was not possible to gather enough data on carnivore mating systems or seasonality to allow meaningful replications of the tests conducted with primates.

References

1. Dixson AF. 1987 Baculum length and copulatory behavior in primates. *Am. J. Primatol.* **60**, 51–60. (doi:10.1002/ajp.1350130107)
2. Campbell CJ, Fuentes A, MacKinnon K, Panger M, Bearder S, editors 2007 *Primates in perspective*. New York: Oxford University Press.
3. Perrin WF, Wursig B, Thewissen JGM, editors 2009 *Encyclopaedia of Marine Mammals*. 2nd edn. London: Academic Press.
4. Yurkowski DJ, Chambellant M, Ferguson SH. 2011 Bacular and testicular growth and allometry in the ringed seal (Pusa hispida): evidence of polygyny? *J. Mammal.* **92**, 803–810. (doi:10.1644/10-MAMM-A-082.1)
5. Fritz SA, Bininda-Emonds ORP, Purvis A. 2009 Geographical variation in predictors of mammalian extinction risk: big is bad, but only in the

tropics. *Ecol. Lett.* **12**, 538–549. (doi:10.1111/j.1461-0248.2009.01307.x)
6. Drummond AJ, Suchard MA, Xie D, Rambaut A. 2012 Bayesian phylogenetics with BEAUti and the BEAST 1.7. *Mol. Biol. Evol.* **29**, 1969–1973. (doi:10.1093/molbev/mss075)
7. Arnold C, Matthews LJ, Nunn CL. 2010 The 10KTrees website: a new online resource for primate phylogeny. *Evol. Anthropol.* **19**, 114–118.
8. Venditti C, Meade A, Pagel M. 2011 Multiple routes to mammalian diversity. *Nature* **479**, 1–18. (doi:10.1038/nature10516)
9. Organ CL, Shedlock AM, Meade A, Pagel M, Edwards SV. 2007 Origin of avian genome size and structure in non-avian dinosaurs. *Nature* **446**, 180–184. (doi:10.1038/nature05621)

Data Sources

Mammals

Baculum Present: Afrosoricida [1,2], Dermoptera [3], Eulipotyphla [1,2].

Baculum Absent: Artiodactyla [3], Cetacea [1,3], Dasyuromorphia [3], Didelphimorphia [3], Diprodontia [3], Hyracoidea [4], Lagomorpha [3] (except *Ochotonia princeps* [5]), Macroscelidea [6,7], Microbiotheria [3], Monotremata [8], Notoryctemorphia [3], Paucituberculata [3], Paramelemorphia [3], Perissodactyla [3], Pholidota [1,2], Probiscoidea [3], Scandentia [3], Sirenia [3] and Tubulidentata [9], Xenarthra [1,2].

Baculum Present & Absent: Carnivora, Chiroptera [10], Primates and Rodentia (all [11], aside from *Hydromys chrysogaster* [12], *Uromys caudimaculatus* [12] and *Notomys alexis* [13]). See individual sections for Primates and Carnivora.

Mammal Data References

1. Perrin WF, Wursig B, Thewissen JGM, editors 2009 *Encyclopaedia of marine mammals.* 2nd ed. London: Academic Press.
2. Yurkowski DJ, Chambellant M, Ferguson SH. 2011 Bacular and testicular growth and allometry in the ringed seal (Pusa hispida): evidence of polygyny? *J. Mammal.* **92**, 803–810. (doi:10.1644/10-MAMM-A-082.1)
3. Martin RD. 2007 The evolution of human reproduction: a primatological perspective. *Yearb. Phys. Anthropol.* **50**, 59–84.
4. Feldhamer GA, Drickamer LC, Vessey SH, Merritt JF, Krajewski C. 2015 *Mammalogy: adaptation, diversity, ecology.* 4th ed. Baltimore, MD: John Hopkins University Press.
5. Weimann B, Edwards MA, Jass CN. 2014 Identification of the baculum in American pika (Ochotona princeps: Lagomorpha) from southwestern Alberta, Canada. *J. Mammal.* **95**, 284–289. (doi:10.1644/13-MAMM-A-165)

6. Olbricht G, Stanley WT. 2009 The topographic distribution of the penis and mammary glands in sengis (Macroscelidea) and its usefulness for taxonomic distributions. *Zoosystematics Evol.* **85**, 297–303.
7. Woodall PF. 1995 The penis of elephant shrews (Mammalia: Macroscelididae). *J. Zool.* **237**, 399–410.
8. Martin RE, Pine RH, DeBlase A., 2011 *A manual of mammalogy: with keys to families of the world*. 3rd ed. Long Grove, IL: Waveland Press.
9. Shoshani J, Goldman CA, Thewissen JGM. 1988 Orycteropus afer. *Mamm. Species*. **300**, 1–8.
10. Hosken DJ, Jones KE, Chipperfield K, Dixson AF. 2001 Is the bat os penis sexually selected? *Behav. Ecol. Sociobiol.* **50**, 450–460. (doi:10.1007/s002650100389)
11. Ramm SA. 2007 Sexual selection and genital evolution in mammals: a phylogenetic analysis of baculum length. *Am. Nat.* **169**, 360–369. (doi:10.1086/510688)
12. Lidicker Jr WZ. 1968 A phylogeny of New Guinea rodent genera based on phallic morphology. *Am. Soc. Mammal.* **49**, 609–643.
13. Breed WG, Leigh CM, Speight N. 2013 Coevolution of the male and female reproductive tracts in an old endemic murine rodent of Australia. *J. Zool.* **289**, 94–100. (doi:10.1111/j.1469-7998.2012.00966.x)

Primates

Baculum Presence:	0 = Absent<br>1 = Present
Intromission Duration:	0 = Short (<180 seconds)<br>1 = Long (>180 seconds)
Mating System:	0 = Monogamy<br>1 = Polygyny<br>2 = Polyandry<br>3 = Polygamy
Breeding Seasonality:	0 = <33% births occur within peak 3 months<br>1 = >33% but <67% births occur within peak 3 months<br>2 = >67% births occur within peak 3 months

Primate Data References

1. Dixson AF. 2012 *Primate Sexuality: Comparative studies of the prosimians, monkeys, apes, and human beings.* 2nd ed. Oxford: Oxford University Press.
2. Hershkovitz P. 1977 *Living New World monkeys (Platyrrhini): volume 1.* Chicago: Chicago Press.
3. Nowak RM. 1999 *Walker's primates of the world.* Baltimore, MD: The John

Hopkins University Press.
4. Dixson AF. 1995 Baculum length and copulatory behaviour in carnivores and pinnipeds (Grand Order Ferae). *J. Zool.* **235**, 67–76.
5. Dixson A, Nyholt J, Anderson M. 2004 A positive relationship between baculum length and prolonged intromission patterns in mammals. *Acta Zool. Sin.* **50**, 490–503.
6. Loy J. 1975 The copulatory behaviour of adult male patas monkeys, Erythrocebus patas. *J. Reprod. Fertil.* **45**, 193–195.
7. Doyle GA, Pelletier A, Bekker T. 1967 Courtship, mating and parturition in the lesser bushbaby (Galago senegalensis moholi) under semi-natural conditions. *Folia Primatol.* **7**, 169–197.
8. Boughner J, Rolian C, editors 2016 *Developmental approaches to human evolution.* New Jersey: Wiley Blackwell.
9. Taub DM. 1982 Sexual behaviour of wild Barbary macaque males (Macaca sylvanus). *Am. J. Primatol.* **2**, 109–113.
10. Harcourt A, Purvis A, Liles L. 1995 Sperm competition: mating system, not breeding season, affects testes size of primates. *Funct. Ecol.* **9**, 468–476. (doi:10.2307/2390011)
11. Campbell CJ, Fuentes A, MacKinnon K, Panger M, Bearder S, editors 2007 *Primates in perspective.* New York: Oxford University Press.
12. Dixson AF, Anderson MJ. 2004 Sexual behavior, reproductive physiology and sperm competition in male mammals. *Physiol. Behav.* **83**, 361–371. (doi:10.1016/j.physbeh.2004.08.022)
13. Fleagle JG. 1999 *Primate adaptation and evolution.* 2nd ed. London: Academic Press.
14. Smuts BB, Cheney DL, Seyfarth RM, Wrangham RW, Struhsaker TT, editors 1987 *Primate societies*. Chicago: The University of Chicago Press.
15. Ramm SA. 2007 Sexual selection and genital evolution in mammals: a phylogenetic analysis of baculum length. *Am. Nat.* **169**, 360–369. (doi:10.1086/510688)
16. Fleagle JG, Mittermeier RA. 1980 Locomotor behavior, body size, and comparative ecology of seven Surinan monkeys. *Am. J. Phys. Anthropol.* **52**, 301–314. (doi:10.1002/ajpa.1330520302)
17. Smith RJ, Cheverud JM. 2002 Scaling of sexual dimorphism in body mass: a phylogenetic analysis of Rensch's rule in primates. *Int. J. Primatol.* **23**, 1095–1135. (doi:10.1023/A:1019654100876)
18. Merker S, Groves CP. 2006 Tarsius lariang: A new primate species from Western Central Sulawesi. *Int. J. Primatol.* **27**, 465–485. (doi:10.1007/s10764-006-9038-z)
19. Wong A. 2014 Covariance between testes size and substitution rates in primates. *Mol. Biol. Evol.* **31**, 1432–1436. (doi:10.1093/molbev/msu091)
20. Gage MJG, Freckleton RP. 2003 Relative testis size and sperm morphometry across mammals: no evidence for an association between sperm competition and sperm length. *Proc. Biol. Sci.* **270**, 625–632. (doi:10.1098/rspb.2002.2258)
21. Soulsbury CD. 2010 Genetic patterns of paternity and testes size in mammals. *PLoS One* **5**. (doi:10.1371/journal.pone.0009581)
22. Kowalewski MM, Garber PA, Cortes-Ortiz L, Urbani B, Youlatos D,

editors 2014 *Howler monkeys: behavior, ecology, and conservation.* New York: Springer.

23. Thorén S, Lindenfors P, Kappeler PM. 2006 Phylogenetic analyses of dimorphism in primates: Evidence for stronger selection on canine size than on body size. *Am. J. Phys. Anthropol.* **130**, 50–59. (doi:10.1002/ajpa.20321)
24. Fietz J, Zischler H, Schwiegk C, Tomiuk J, Dausmann KH, Ganzhorn JU. 2000 High rates of extra-pair young in the pair-living fat-tailed dwarf lemur, Cheirogaleus medius. *Behav. Ecol. Sociobiol.* **49**, 8–17. (doi:10.1007/s002650000269)
25. Kay RF, Plavca JM, Glander KE, Wright PC. 1988 Sexual selection and canine dimorphism in New World monkeys. *Am. J. Phys. Anthropol.* **77**, 385–397. (doi:10.1002/ajpa.1330770311)
26. Rode-Margono EJ, Nekaris KAI, Kappeler PM, Schwitzer C. 2015 The largest relative testis size among primates and aseasonal reproduction in a nocturnal lemur, Mirza zaza. *Am. J. Phys. Anthropol.* **158**, 165–169. (doi:10.1002/ajpa.22773)
27. Marolf B, McElligott AG, Muller AE. 2007 Female social dominance in two Eulemur species with different social organisations. *Zoo Biol.* **26**, 201–214. (doi:10.1002/zoo)
28. Fuentes A. 1998 Re-evaluating primate monogamy. *Am. Anthropol.* **100**, 890–907.
29. Gouzoules S. 1984 Primate mating systems, kin associations, and cooperative behavior: Evidence for kin recognition? *Am. J. Phys. Anthropol.* **27**, 99–134. (doi:10.1002/ajpa.1330270506)
30. Robbins M. 1999 Male mating patterns in wild multimale mountain gorilla groups. *Anim. Behav.* **57**, 1013–1020. (doi:10.1006/anbe.1998.1063)
31. Nievergelt CM, Mutschler T, Feistner ATC, Woodruff DS. 2002 Social system of the Alaotran gentle lemur (Hapalemur griseus alaotrensis): Genetic characterization of group composition and mating system. *Am. J. Primatol.* **57**, 157–176. (doi:10.1002/ajp.10046)
32. Dixson AF, Anderson M. 2001 Sexual selection and the comparative anatomy of reproduction in monkeys, apes, and human beings. *Annu. Rev. Sex Res.* **12**, 121–144. (doi:10.1080/10532528.2001.10559796)
33. Zinner D, Hilgartner RD, Kappeler PM, Pietsch T, Ganzhorn JU. 2003 Social organisation of Lepilemur ruficaudatus. *Int. J. Primatol.* **24**, 869–888. (doi:10.1023/A)
34. Müller E, Thalmann U. 2000 Origin and evolution of primate social organisation: a reconstruction. *Biol. Rev. Camb. Philos. Soc.* **75**, 405–35. (doi:10.1111/j.1469-185X.2000.tb00050.x)
35. Kappeler PM, Van Schaik CP. 2002 Evolution of primate social systems. *Int. J. Primatol.* **23**, 707–740. (doi:10.1023/A:1015520830318)
36. Nekaris KAI. 2003 Spacing system of the Mysore slender loris (Loris lydekkerianus lydekkerianus). *Am. J. Phys. Anthropol.* **121**, 86–96. (doi:10.1002/ajpa.10189)
37. Harcourt AH, Harvey PH, Larson SG, Short RV. 1981 Testis weight, body weight and breeding system in primates. *Nature.* **293**, 55–57. (doi:10.1038/293055a0)

38. Weckerly FW. 1998 Sexual-size dimorphism: influence of mass and mating systems in the most dimorphic mammals. *J. Mammal.* **79**, 33–52. (doi:10.1126/science.95.2469.427-b)
39. Dammhahn M, Kappeler PM. 2005 Social system of Microcebus berthae, the world's smallest primate. *Int. J. Primatol.* **26**, 407–435. (doi:10.1007/s10764-005-2931-z)
40. Génin F. 2008 Life in unpredictable environments: First investigation of the natural history of Microcebus griseorufus. *Int. J. Primatol.* **29**, 303–321. (doi:10.1007/s10764-008-9243-z)
41. Rode EJ, Nekaris KA-I, Markolf M, Schliehe-Diecks S, Seiler M, Radespiel U, Schwitzer C. 2013 Social organisation of the northern giant mouse lemur Mirza zaza in Sahamalaza, north western Madagascar, inferred from nest group composition and genetic relatedness. *Contrib. to Zool.* **82**, 71–83.
42. Wiens F, Zitzmann A. 2003 Social structure of the solitary slow loris *Nycticebus coucang* (Lorisidae). *J. Zool.* **261**, 35–46. (doi:10.1017/S0952836903003947)
43. Hager R, Welker C, 2001 Female dominance in African lorises (Otolemur garnettii). *Folia Primatol.* **72**, 48–50.
44. Atmoko SSU, Setia TM, Goossens B, James SS, Knott CD, Morrogh-Bernard HC, van Schaik CP, van Noordwijk MA. 2008 Orangutan mating behaviour and strategies. In Wich SA, Atmoko SSU, Setia TM, van Schaik CP, editors. *Orangutans: geographic variation in behavioural ecology and conservation.* Oxford: Oxford University Press. p. 235-244
45. Galdikas BMF. 1985 Adult male sociality and reproductive tactics among orangutans at Tanjung Puting. *Folia Primatol.* **45**, 9–24.
46. Boinski S. 1987 Mating patterns in squirrel monkeys (Saimiri oerstedi): implications for seasonal sexual dimorphism. *Behav. Ecol. Sociobiol.* **21**, 13–21.
47. Merker S. 2010 The population ecology of Dian's tarsier. In Gursky-Doyen S, Supriatna J, editors. *Indonesian primates*. New York: Springer. p. 371–382.
48. Dagosto M, Gebo DL, Dolino C. 2001 Positional behaviour and social organization of the Philippine tarsier (Tarsius syrichta). *Primates* **42**, 233–243. (doi:10.1007/BF02629639)
49. Zhou Q-H, Luo B, Huang C-M. 2013 Attacks on adult females with infants by non-resident males in the François langur. *Zool. Res.* **34**, E9–E12. (doi:10.3724/SP.J.1141.2013.E01E09)
50. Vasey N. 2007 The breeding system of wild red ruffed lemurs (Varecia rubra): a preliminary report. *Primates* **48**, 41–54. (doi:10.1007/s10329-006-0010-5)

<u>Carnivores</u>

<u>Baculum Presence:</u> 0 = Absent
1 = Present

Intromission Duration: 0 = Short (<180 seconds)
1 = Long (>180 seconds)

Carnivore Data References

1. Dixson AF. 1995 Baculum length and copulatory behaviour in carnivores and pinnipeds (Grand Order Ferae). *J. Zool.* **235**, 67–76.
2. Dixson AF, Nyholt J, Anderson M. 2004 A positive relationship between baculum length and prolonged intromission patterns in mammals. *Acta Zool. Sin.* **50**, 490–503.
3. Perrin WF, Wursig B, Thewissen JGM, editors. 2009 *Encyclopaedia of marine mammals*. 2nd ed. London: Academic Press.
4. Ewer RF, 1973 *The carnivores*. New York: Cornell University Press.
5. Drea CM, Place NJ, Weldele ML, Coscia EM, Licht P, Glickman SE. 2002 Exposure to naturally circulating androgens during foetal life incurs direct reproductive costs in female spotted hyenas, but is prerequisite for male mating. *Hungarian Q.* **49**, 1981–1987. (doi:10.1098/rspb.2002.2109)
6. Martin RD. 2007 The evolution of human reproduction: a primatological perspective. *Yearb. Phys. Anthropol.* **50**, 59–84.
7. Baryshnikov GF, Bininda-Emonds ORP, Abramov AV. 2003 Morphological variability and evolution of the baculum (os penis) in Mustelidae (Carnivora). *J. Mammal.* **84**, 673–690. (doi:10.1644/1545-1542(2003)084<0673:MVAEOT>2.0.CO;2)
8. Mills MGL. 1983 Mating and denning behaviour of the brown hyaena *Hyaena brunnea* and comparisons with other Hyaenidae. *Zeitschrift für Tierzüchtung und Züchtungsbiologie* **63**, 331–342. (doi:10.1111/j.1439-0310.1983.tb00748.x)
9. Larivière S, Ferguson SH. 2002 On the evolution of the mammalian baculum: vaginal friction, prolonged intromission or induced ovulation? *Mamm. Rev.* **32**, 283–294. (doi:10.1046/j.1365-2907.2002.00112.x)
10. Dyck MG, Bourgeois JM, Miller EH. 2004 Growth and variation in the bacula of polar bears (*Ursus maritimus*) in the Canadian Arctic. *J. Zool.* **264**, 105–110. (doi:10.1017/S0952836904005606)
11. Roberts, M, Kessler DS. 1979 Reproduction in red pandas,. *J. Zool.* **188**, 235–249. (doi:10.1111/j.1469-7998.1979.tb03402.x)
12. Hass CC, Roback JF. 2000 Copulatory behaviour of White-Nosed Coatis. *Southwest. Nat.* **45**, 329–331. (doi:10.1894/0038-4909-55.2.310)
13. Soulsbury CD. 2010 Genetic patterns of paternity and testes size in mammals. *PLoS One* **5**. (doi:10.1371/journal.pone.0009581)
14. Ferguson SH, Larivière S. 2004 Are long penis bones an adaption to high latitude snowy environments? *Oikos* **105**, 255–267. (doi:10.1111/j.0030-1299.2004.13173.x)
15. Iossa G, Soulsbury CD, Baker PJ, Harris S. 2008 Sperm competition and the evolution of testes size in terrestrial mammalian carnivores. *Funct. Ecol.* **22**, 655–662. (doi:10.1111/j.1365-2435.2007.0)
16. Johnson DDP, Macdonald DW, Dickman AJ. 2000 An analysis and

review of models of the sociobiology of the Mustelidae. *Mamm. Rev.* **30**, 171–196. (doi:10.1046/j.1365-2907.2000.00066.x)
17. McNab BK. 2000 The standard energetics of mammalian carnivores: Felidae and Hyaenidae. *Can. J. Zool.* **78**, 2227–2239. (doi:10.1139/z00-167)
18. Mills MGL. 1982 Notes on age determination, growth and measurements of brown hyaenas *Hyaena brunnea* from the kalahari gemsbok national park. *Koedoe.* **25**, 55–61.
19. Wroe S, McHenry C, Thomason J. 2005 Bite club: comparative bite force in big biting mammals and the prediction of predatory behaviour in fossil taxa. *Proc. R. Soc. B* **272**, 619–625. (doi:10.1098/rspb.2004.2986)
20. Ramm SA. 2007 Sexual selection and genital evolution in mammals: a phylogenetic analysis of baculum length. *Am. Nat.* **169**, 360–369. (doi:10.1086/510688)
21. Gage MJG, Freckleton RP. 2003 Relative testis size and sperm morphometry across mammals: no evidence for an association between sperm competition and sperm length. *Proc. Biol. Sci.* **270**, 625–632. (doi:10.1098/rspb.2002.2258)
22. Campagna C, Le Boeuf BJ. 1988 Reproductive behaviour of Southern Sea Lions. *Behavior* **104**, 233–261. (doi:10.1163/156853988X00539)
23. Lanier DL, Dewsbury DA. 1976 A quantitative study of copulatory behaviour of large Felidae. *Behav. Processes* **1**, 327–333. (doi:10.1016/0376-6357(76)90014-0)
24. Lindeberg H. 2008 Reproduction of the female ferret (Mustela putorius furo). *Reprod. Domest. Anim.* **43**, 150–156. (doi:10.1111/j.1439-0531.2008.01155.x)
